# Cyclodextrin nanosponge for the GSH-mediated delivery of Resveratrol in human cancer cells

**DOI:** 10.7150/ntno.53888

**Published:** 2021-01-21

**Authors:** Marco Palminteri, Nilesh Kumar Dhakar, Alessandra Ferraresi, Fabrizio Caldera, Chiara Vidoni, Francesco Trotta, Ciro Isidoro

**Affiliations:** 1 Laboratory of Molecular Pathology, Department of Health Sciences, Università del Piemonte Orientale “A. Avogadro”, Novara, Italy.; 2 Department of Chemistry, University of Turin, via P. Giuria 7, 10125, Turin, Italy.

**Keywords:** Resveratrol, glutathione, breast cancer, ovarian cancer, β-cyclodextrin nanosponge

## Abstract

Smart drug delivery systems are required for the site-specific drug targeting to enhance the therapeutic efficiency of a drug. Resveratrol (RV) is a polyphenolic compound with anti-cancer activity. However, its poor aqueous solubility and non-selectivity are the major challenges for its employment in cancer therapy. In this work, we present the synthesis of RV-loaded glutathione responsive cyclodextrin nanosponges (RV-GSH-NSs) to improve the therapeutic efficiency and selective delivery of RV. The drug loading and encapsulation efficiency were 16.12% and 80.64%, respectively. The *in vitro* release profile confirmed that RV release was enhanced in response to external glutathione (GSH). Nude NSs were not toxic *per se* to human fibroblasts when administered for up to 72 h at the highest dose. Cell internalization studies confirmed that RV-GSH-NSs were preferentially up-taken by tumor cells compared to non-tumorigenic cells. Accordingly, RV showed selective toxicity to cancer cells compared to normal cells. GSH depletion by buthionine sulfoximine, a potent inhibitor of its synthesis, reflected in a significant decrease of the NSs accumulation, and consequently resulted in a drastic reduction of RV-mediated toxic effects in cancer cells. These findings demonstrate that GSH- responsive NSs represent an effective delivery system for targeting cancer cells by harnessing the differential tumor characteristics in terms of redox status in parallel with the limitation of side effects toward normal cells.

## Introduction

Breast and ovarian cancers are among the most frequently diagnosed and deadly gynecological cancers 1. Chemotherapy, in association or not with surgery, is the most common therapeutic option for the treatment of these cancers. However, chemotherapeutic drugs are associated with several drawbacks such as low selectivity, low aqueous solubility, and low bioavailability 2, 3. To overcome these challenges new therapeutic approaches are required, and smart drug delivery systems could represent a valuable tool. Resveratrol (RV) is a natural polyphenolic compound that can be obtained from various natural sources such as grapes, peanuts, mulberries, and Japanese knotweed 4. RV provides a wide range of preventive and therapeutic effects against different types of cancer, as testified by the increasing number of ongoing clinical trials that are screening its effectiveness as anti-cancer 5. As a matter of fact, RV has shown the ability to reduce tumor growth *in vivo* 6-8, and this anti-cancer activity has been linked to epigenetic mechanisms 9, 10, autophagy 11-13, apoptosis 14, 15, induction of senescence 16, 17, interruption of the crosstalk between cancer cells and stromal fibroblasts 18, among others. However, low aqueous solubility, chemical instability, and non-selectivity are the major limitations for the employment of RV in cancer therapy in humans 19-21. Therefore, RV formulations with increased bioavailability specifically at the tumor site are needed.

Smart drug delivery systems such as liposomes, dendrimers, stimuli-responsive polymeric nanoparticles, and micelles are extensively used to improve the safety and efficacy of the administered drug molecules 22, 23. Cyclodextrin-based nanocarriers can form inclusion complexes with therapeutics molecules increasing their aqueous solubility and stability, and therefore represent also a valid system for drug delivery 24, 25. Cyclodextrin nanosponges (NSs) are hyper-crosslinked polymers featuring high drug loading and solubilization efficiency. Anti-cancer drugs used as the guest molecules in NSs include paclitaxel 26, camptothecin 27, erlotinib 28, oxy-resveratrol 29, and tamoxifen 30. However, non-selectivity of the developed formulation still limits the pharmacologic efficacy and remains a challenging task.

Stimuli-responsive drug delivery systems offer the advantage of site-specific drug delivery and release in response to tumor chemical characteristics such as pH, intracellular enzymes, and redox gradient 31-33. For instance, the concentration of glutathione (GSH) is higher in tumor cells (0.5-10 mM) compared to the normal cells (2-20 µM) 34. This significant difference in terms of GSH concentration has prompt the design of GSH-responsive nanocarriers for tumor-targeted delivery of the drugs 35. The presence of disulfide groups in the NSs promotes the release of drug molecules in response to the intracellular GSH concentrations. Daga and co-workers demonstrated the *in vitro* anti-cancer efficiency of doxorubicin (DOX)-loaded GSH-responsive NSs in different cancer cells 36. Moreover, *in vivo* studies suggested a prolonged plasma circulation time of the DOX-GSH-NSs compared to free DOX 36. GSH-responsive cyclodextrin NSs loaded with anticancer drug have been shown to kill preferentially cancer cells highly expressing GSH 37.

In the present study, we developed the GSH-responsive NSs (GSH-NSs) for the tumor-specific delivery of RV. We validated *in vitro* the selectivity of the targeting of our delivery system by demonstrating the preferential uptake of RV-GSH-NSs in cancer cells rather than in normal cells. We prove that this differential internalization reflects in a selective cytotoxicity towards cancer cells highly expressing GSH, as indicated by the fact that GSH depletion abrogates RV-GSH-NSs toxicity. Further, we show that chronic administration of nude GSH-NSs at high concentration is not toxic to normal fibroblasts.

## Materials and Methods

The β-cyclodextrin (β-CD) was a kind gift from Roquette Italia (Cassano Spinola, Italy). Resveratrol, pyromellitic dianhydride, 2-hydroxyethyl disulfide, and glutathione were purchased from Sigma-Aldrich (St. Louis, MO, USA). Unless otherwise specified, all other chemicals were of analytical grade.

### Synthesis of the GSH-NSs

Glutathione-responsive β-CD nanosponges (GSH-NSs) were prepared by a method developed by our group earlier 38. Briefly, 2.0 g (1.76 mmol) of anhydrous β-CD was dissolved in 8 mL of DMSO with continuous stirring until a clear solution is formed. Later, 0.200 g (1.29 mmol) of 2-hydroxyethyl disulfide and 2.0 mL (14.35 mmol) of triethylamine was added as a catalyst. Finally, 5.5 g (24.48 mmol) of pyromellitic dianhydride was added to the solution with vigorous stirring to carry out the reaction. Gel-like mass was obtained within a few minutes which was incubated for the next 24 hours at room temperature to complete the reaction. At the end of the reaction, a solid monolith block of GSH-NSs was crushed to obtain a coarse powder followed by extensive washing with water and acetone. The prepared GSH-NSs were purified by Soxhlet extraction with acetone for a period of approximately 24 hours and air-dried at room temperature. GSH-NSs were kept in a desiccator for further use. The sulfur content within the GHS-NSs was determined by elemental analysis (Thermo Electron Corporation Flash EA 1112 series CHNS-O Analyzer) using an equal amount of V_2_O_5_ as a catalyst (2.5 mg).

### Preparation of RV and Coumarin-6 loaded GSH-NSs

Before performing drug loading, nanosuspension of GSH-NSs (10 mg/mL in water or saline) was prepared by high shear homogenizer (Ultraturrax^®^, IKA, Konigswinter, Germany) for 10-15 min at 24,000 rpm followed by high-pressure homogenization (HPH) for 1.5 hours at a back pressure of 500 bar using an EmulsiFlex C5 instrument (Emulsiflex C5, Avestin, USA). Later, nanosuspension was dialyzed for a few minutes.

RV-loaded GSH-NSs were prepared by adding RV in a different weight ratio of 1:2, 1:4, and, 1:6 (w/w; drug: nanosponge) in a nanosuspension of GSH-NSs (10 mg/mL). Later, samples were sonicated for 20 minutes followed by continuous stirring for 24 hours in dark. Samples were subjected to mild centrifugation and supernatant was collected followed by dialysis in water for a few minutes to remove the unloaded drug. RV-loaded GSH-NSs were freeze-dried and stored in a desiccator for further characterization. Fluorescent NSs were prepared in a similar manner by taking NS suspension (10 mg/mL) in saline with 0.1 mg/mL coumarin-6 (C-6).

### Quantitative determination of the RV

The concentration of RV was quantified using an HPLC system (PerkinElmer, Waltham, USA) equipped with a UV detector (Flexar UV/Vis LC spectrophotometer). We used a reversed phase Phenomenex C18 analytical column (4.6 mm × 250 mm, 5 µm) with a mobile phase consisted acetic acid in methanol (0.5 %, v/v) and water (52:48, v/v). The degassed mobile phase was passed through the column with a flow rate and injection volume of 1 mL/min and 20 µL, respectively at an absorption maxima of 305 nm and retention time of 6.9 minutes 39. A calibration curve was recorded at the concentration of 2-10 µg/mL and a good correlation coefficient of 0.9997 was observed.

### Determination of the drug loading and encapsulation efficiency

Freeze-dried RV-GSH-NSs were suspended into a vial containing 2 mL of methanol and sonicated for 2 hours. Later, it was centrifuged, and the supernatant was analyzed after appropriate dilution with the mobile phase.

The following equation was used to determine drug loading and encapsulation efficiency:

Drug Loading (% DL) = [Entrapped RV/Weight of GSH-NSs]*100

Encapsulation Efficiency (% EE) = [Entrapped RV/Total RV]*100

### Particle size, polydispersity index (PDI), and zeta potential of RV-GSH-NSs

The mean particle size and polydispersity index of blank GSH-NSs, RVGSH-NSs, and C-6 loaded GSH-NSs were determined by dynamic light scattering with the help of Malvern Zetasizer Nano (Worcestershire, UK) after suitable dilution with HPLC grade water. An additional electrode was placed inside the zetasizer to determine the zeta potential of all the samples by measuring the electrophoretic mobility at room temperature.

### Thermal analysis

Differential scanning calorimetry (DSC) measurements were performed using a TA instruments Q200 DSC (New Castle, DE, USA) at a temperature range of 30-300 °C with a scanning rate of 10 °C/min. Freeze-dried samples (2-3 mg) were placed inside the standard aluminum pan and an empty pan was used as a standard. All the samples were analyzed under a nitrogen purge of 50 mL/min.

### Fourier Transform Infrared Spectroscopy (FTIR)

The FTIR spectra of RV, Blank GSH-NSs, and RV-loaded GSH-NSs were obtained by PerkinElmer 100 FTIR to determine the interaction of RV with GSH-NSs. The spectra were recorded on attenuated total reflectance (ATR) assembly in a range of 4000-650 cm^-1^ at a resolution of 4 cm^-1^ and analyzed on spectrum software.

### Powder X-ray diffraction studies

Malvern Panalytical X'Pert diffractometer (Worcestershire, UK) was used to record the diffraction pattern of RV, Blank GSH-NSs, and RV-loaded GSH-NSs. Cu Kα1 was employed as a radiation source and data acquisition was carried out at the diffraction angle of 5-45° 2θ at a step size of 0.015°.

### Determination of the morphology

Transmission electron microscopy (TEM) and field emission scanning electron microscopy (FE-SEM) studies were performed to determine the particle shape and size. Aqueous nanosuspension of samples were placed on the aluminum stub with the help of a bio-adhesive carbon tape and air-dried followed by sputter-coating with gold to analyze on FE-SEM (ZEISS supra 40, Oberkochen, Germany). For TEM (JEOL JEM 3010, MA, USA) studies aqueous nanosuspension of samples were sprayed on Formvar coated copper grid and air-dried before image acquisition.

### *In vitro* drug release

*In vitro* release study of RV from the GSH-NSs was carried out in phosphate buffer pH 7.4 by membrane diffusion method using a dialysis membrane cut off size of 12 kDa. A known quantity of RV-GSH-NSs (10 mg) was suspended in 1 mL of phosphate buffer solution. The suspension was filled in the dialysis bag and placed inside a vial containing 10 mL of a phosphate buffer solution with continuous stirring of 50 rpm at 37 ± 0.5 °C. The effect of GSH on the drug release was also studied by adding 10 mM GSH and 20 mM GSH in the dissolution media. The aliquots (1 mL) were withdrawn at different time intervals and replaced with the same amount of respective fresh phosphate buffer. Later, all the samples were filtered with a 0.4 µm syringe filter and analyzed on HPLC after suitable dilution with the mobile phase.

### Cell culture

All cell lines were obtained from ATCC (Manassas, Virginia, USA) and maintained under standard culture conditions (37°C, 5% CO_2_). SKOV3 and OVCAR3 human ovarian cancer cell lines were cultured in RPMI 1640 medium supplemented with 10% heat-inactivated fetal bovine serum (FBS), 1% Glutamine and 1% Penicillin/Streptomycin solution. MDAMB231 human triple-negative breast cancer cells and human fibroblasts were cultured in DMEM medium supplemented with 10% heat-inactivated fetal bovine serum (FBS), 1% Glutamine and 1% Penicillin/Streptomycin solution. MCF10A human mammary epithelial cell line was cultured in DMEM-F12 (1:1) medium supplemented with 15% heat-inactivated horse serum, 1% Glutamine and 1% Penicillin/Streptomycin solution, 500 µg/ml hydrocortisone, 100 ng/ml cholera toxin, 10 µg/ml insulin and 20 ng/ml EGF.

### Intracellular accumulation of the GSH-NSs

Cells were plated on sterile coverslips and then incubated with C-6-loaded GSH-NSs for the indicated time points. Coverslips were promptly observed under the fluorescence microscope. To confirm that cell uptake and intracellular accumulation were regulated by intracellular glutathione, the cells were depleted of intracellular GSH by pre-treatment for 16 h with buthionine sulfoximine (BSO; cod. 004CA14484, Cayman), a potent inhibitor of the enzyme gamma-glutamyl-cysteine synthetase. BSO was dissolved in sterile water and used at 1 mM final concentration.

### Cell tracker staining

Cells plated on sterile coverslips and treated with the RV-GSH-NSs as indicated were labeled with the fluorescent dye Cell Tracker (Cell Tracker Blue-CMAC 7-amino-4-chloromethylcoumarin; incubation for 30 min in serum-free media at 37 °C followed by additional 30 min in complete media at 37 °C; cod. C2110, Life Technologies) as previously reported 40. At the end of treatment, fluorescence stained coverslips were promptly observed under the fluorescence microscope.

### Propidium iodide staining

Cells plated on sterile coverslips were treated as indicated. Necrotic cells were detected by using 0.2 μg/ml propidium iodide (PI; incubation for 10 min in the dark at 37 °C; cod. P4170, Sigma Aldrich). At the end of treatment, fluorescence stained coverslips were promptly observed under the fluorescence microscope.

### Imaging acquisition and analysis

Fluorescence images were acquired at the multi-channel fluorescence microscope (Leica Microsystems, Wetzlar, Germany; DMI6000). For each experimental condition, at least three slides were prepared in separate experiments and five to ten microscopic fields randomly chosen were imaged by two independent investigators unaware of the treatment. At least 100 to 150 cells were considered in total. Quantification of fluorescence intensity was performed with the software ImageJ. Representative images of selected fields are shown.

### Western blotting

Cells were seeded in petri dishes at the cell density of 40,000-50,000 cell/cm^2^ and cultured up to approximately 80% of confluence. Cells were washed once with PBS 1X and harvested with lysis buffer containing 0.2% NaDOC and supplemented with protease inhibitor cocktail and phosphatases inhibitors (50 mM NaF, 1 mM Na_3_VO_4_). Bradford assay was used to measure the protein content. Equal amounts of cell homogenates (30 μg) were denatured at 95°C for 10 min, resolved by SDS-PAGE and thereafter blotted onto PVDF membrane. For detection of GSH by western blotting, we performed a 20% PAGE under non-reducing conditions (avoiding the use of SDS and of DTT, as recommended by the manufacturer). The membranes were blocked with 5% non-fat dry milk + 0.2% Tween for 1 hour at room temperature and thereafter incubated with primary antibodies overnight at 4°C. The following day, the membranes were washed and incubated with the secondary antibody (Goat anti-mouse (cod. 170-6516) or Goat anti-rabbit (cod. 170-6515); Bio-Rad) for 1 hour at room temperature. The membranes were washed, and the bands were detected by using Enhanced Chemiluminescence reagents (ECL; cod. NEL105001EA; PerkinElmer) and imaged with the VersaDOC Imaging System. For loading control, the membranes were re-probed with housekeeping markers (i.e. β-tubulin, β-actin, GAPDH). The intensity of the bands was estimated by densitometry using Quantity One software.

### Antibodies

The following antibodies were used: mouse anti-β-actin (1:2000, cod. A5441, Sigma Aldrich), rabbit anti-GAPDH (1:1000, cod. G9545, Sigma Aldrich). mouse anti-caspase 8 (1:500, cod. 9746, Cell Signaling), mouse anti-β-tubulin (1:1000, cod. T5201, Sigma Aldrich), mouse anti-glutathione (1:1000, cod. MA17620, Life Technologies), mouse anti-caveolin-1 (1:1000, cod. 610406, BD).

### Statistical analysis

Statistical analysis was performed with GraphPad Prism 5.0 software. Bonferroni's multiple comparison test after one-way or two-way ANOVA analysis (unpaired, two-tailed) were employed. Significance was considered as follow: **** p< 0.0001; ***p < 0.001; **p < 0.01; *p < 0.05. All data are reported as average ± S.D.

## Results

### Physicochemical Characterization of the RV-GSH-NSs

GSH-responsive β-CD-NSs were prepared from pyromellitic dianhydride and 2-hydroxyethyl disulfide as the crosslinkers. A schematic of the structure of the GSH-NSs is shown in Supplementary Figure S1. After complete purification, we first performed an elemental analysis to determine the presence of sulfur in the blank GSH-NSs. The carbon and hydrogen contents were 54.42% and 5.33%, respectively as confirmed by CHNS analysis. Moreover, the sulfur content in the GSH-NSs was 0.75%. However, the sulfur content was lower than the theoretical value of 0.97%. The lower sulfur content could be attributed to the low reactivity of 2-hydroxyethyl disulfide as a crosslinking agent compared to β-CD. Furthermore, elemental analysis results are in agreement with the previously reported data 38. The solubilization of RV in the presence of GSH-NSs was studied to confirm the enhancement in the aqueous solubility which showed more than four-fold higher solubilization (201 µg/mL) in water compared to free RV (46 µg/mL). The increase in the solubility could be attributed to the presence of multiple cavities of the CDs in the polymeric matrix of the GSH-NSs. The loading of RV with GSH-NSs was carried out by taking different weight ratios of 1:2, 1:4, and 1:6 (w/w; RV-GSH-NSs), respectively. The RV loading was 9.95% at 1:2 w/w which increased significantly to 16.12% at 1:4 w/w. However, RV loading decreased to 13.72% at 1:6 w/w, possibly due to the saturation of the RV into the GSH-NSs. As per the drug loading data, RV-GSH-NSs (1:4 w/w) were chosen to carry out further studies. In Supplementary Table 1, the particle size, polydispersity index (PDI), zeta potential, and encapsulation efficiency of GSH-NSs formulations are reported. The particle size of blank GSH-NSs was less than 200 nm, and zeta potential was high enough to ensure the physical stability of nano-formulation in order to avoid the agglomeration of the colloidal NS particles. The physical interaction of RV with GSH-NSs was evaluated by FTIR, DSC, and PXRD studies. The FTIR spectra of blank GSH-NSs, RV, and RV-GSH-NSs are shown in Supplementary Figure S2(A). The FTIR spectrum of RV showed characteristic peaks at 3210 cm^-1^ due to stretching of the O-H group, followed by C-H stretching of phenyl ring at 3017 cm^-1^, C=C stretching at 1608 cm^-1^, and O-H bending at 1325 cm^-1^. It was also observed that RV encapsulation within GSH-NSs led to the change and shift in the characteristic vibrations of the RV. This change in the FTIR spectrum of RV could be due to the interaction of the drug with GSH-NSs.

DSC studies were performed to confirm the encapsulation of the RV within the GSH-NSs. No endothermic transitions were observed with blank GSH-NSs which confirm the stability of the NSs at the tested temperature range as shown in Supplementary Figure S2(B). However, RV showed an endothermic peak at 268.32 °C, which corresponds to its melting point. The endothermic melting peak RV was masked in case of RV-GSH-NSs which could be attributed to the encapsulation and subsequent dispersion in the molecular state thus unable to crystallize.

Supplementary Figure S2(C) represents the diffraction pattern of blank GSH-NSs, RV, and RV-GSH-NSs, respectively. Diffractogram of RV showed characteristic diffraction peaks at a 2θ angle of 6.59, 16.40, 19.22, 20.36, 22.40, 23.64, 24.15, and 28.35 which confirms its crystalline nature. However, RV-GSH-NSs and blank GSH-NSs do not exhibit any intense peak similar in their respective PXRD pattern. The loss in the crystallinity of the RV confirmed the molecular dispersion into the GSH-NSs due to the successful encapsulation within the nanosponge matrix. These findings were consistent with previously reported data related to RV and other NSs 26, 27, 41.

The morphology and size of the RV-GSH-NSs were determined by TEM and FE-ESM as shown in Supplementary Figure S2(D). TEM and FE-SEM images of RVGSH-NSs confirmed that the particles were uniform and in the nanoscale range. The particle size obtained with TEM and FE-SEM were also in agreement with the DLS results (180-200 nm).

### *In vitro* release profile of RV from GSH-NSs

**Figure 1:**
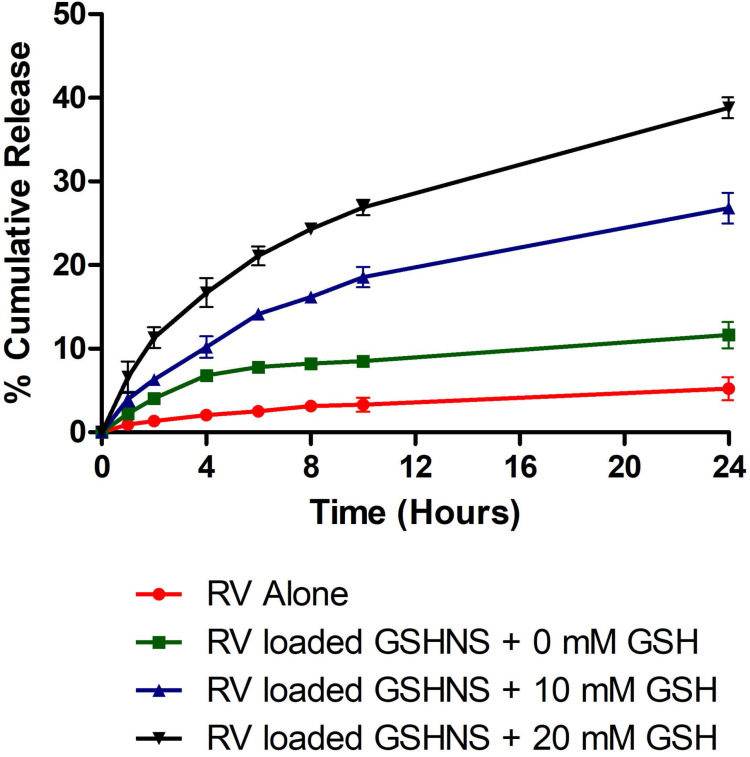
** The release profile of the RV and RV-loaded-GSH-NSs** (dissolution medium: Phosphate Buffer pH 7.4 Solution, temperature: 37 ± 0.5 °C, rotation speed: 50 rpm).

The *in vitro* release profile of RV-GSH-NSs was investigated with and without GSH as shown in the Figure 1. It was observed that a slow and consistent release of RV was obtained in the response to external GSH concentration with no initial burst effect. Moreover, RV-GSH-NSs exhibited higher drug release compared to RV alone. After 24 hours, RV-GSH-NSs showed almost two-fold higher drug release compared to RV alone. A higher release profile of RV might be due to the enhanced solubilization potential of the GSH-NSs. Furthermore, RV-GSH-NSs showed more than five-fold higher drug release compared to free RV in the presence of 10 mM GSH that was further enhanced to eight-fold with 20 mM GSH. This behavior of the GSH-NSs confirmed the GSH responsiveness of the NSs. The presence of GSH in the release media causes a rapid breakdown of the nanocarrier which allows the higher release of RV.

### GSH-NSs preferentially enter in cancer cells compared to their benign counterparts

**Figure 2:**
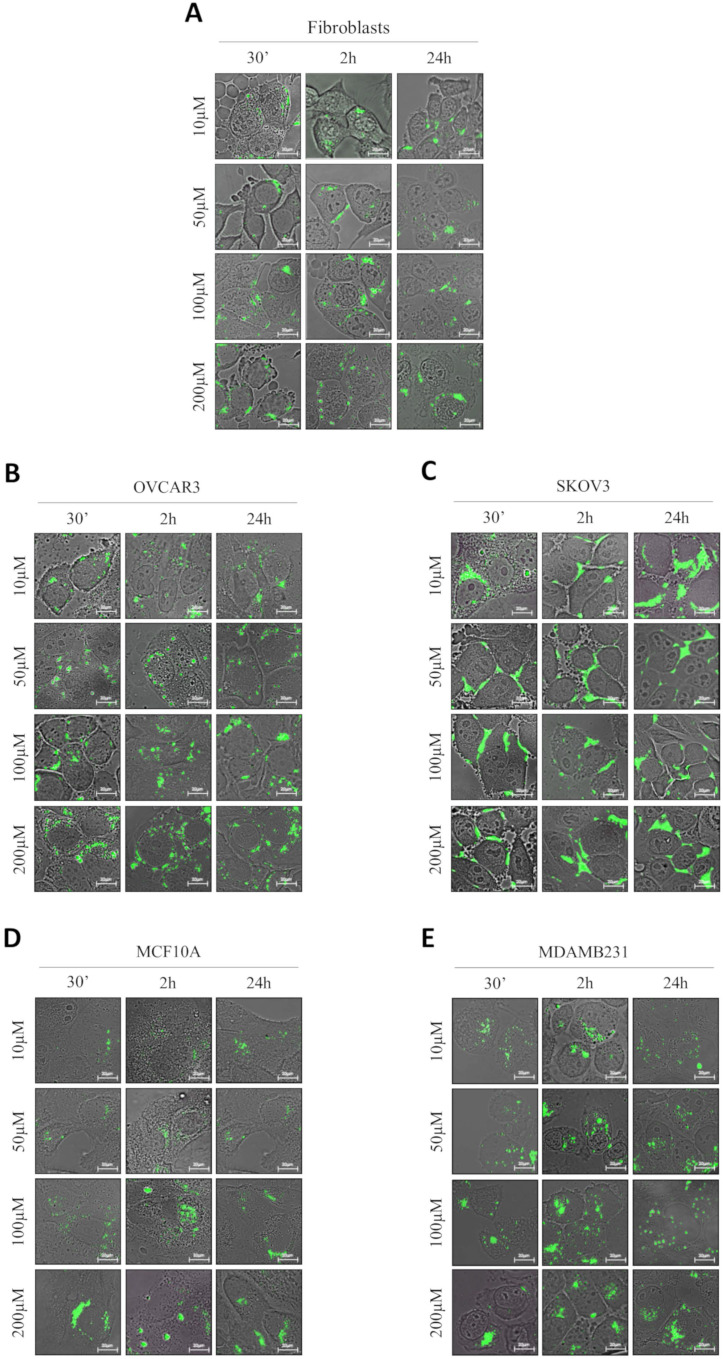
** GSH-NSs preferentially enter and accumulate in cancer cells compared to normal cells.** Cells were plated on sterile coverslips and then incubated with increasing concentrations of C-6-GSH-NSs for the indicate time points. C-6-GSH-NSs exhibit an intrinsic green fluorescence and share with the RV-GSH-NSs the same methods of synthesis and the same dimensions. So, internalization and intracellular distribution of C-6-GSH-NSs can be assumed similar to those of RV-GSH-NSs. Cell internalization studies were performed on normal fibroblasts (**A**), on two ovarian cancer models, OVCAR3 cells (**B**) and SKOV3 cells (**C**), on a non-tumorigenic breast model, MCF10A (**D**) and a breast cancer cell line, MDAMB231 (**E**).

We addressed whether the responsiveness of NSs to GSH reflects in a differential uptake by cancer cells and benign cells. For this purpose, we have employed normal fibroblasts, two ovarian cancer cell models (OVCAR3 and SKOV3 cells) differing in the genetic background, and a breast cancer model (MDAMB231 cells) and its non-tumorigenic counterpart (MCF10A cells). As shown in Figure 2, we employed four concentrations of GSH-NSs (ranging from 10 up to 200 µM) at three different time-points (30 minutes, 2 hours and 24 hours).

To test the internalization of RV-GSH-NSs, we employed C-6-loaded GSH-NSs that have intrinsic green fluorescence and shares with the RV-GSH-NSs the same methods of synthesis and the same dimensions. The quantification of the internalization rate in the different cell models is reported in Supplementary Figure S3. Our data show that OVCAR3 cells record the highest internalization rate at each time point considered, followed by MDAMB231 cells, which exhibit an uptake capacity about 25% less than OVCAR3 cells. Moreover, we noticed that MDAMB231 cells showed a decrease of C-6-GSH-NSs accumulation rate after 24 hours, possibly due to extrusion. Further investigations are needed to prove this speculation.

On the other hand, we found that fibroblasts internalize C-6-GSH-NSs about 4-times less than OVCAR3 cells and 3-times less than MDAMB231 cells. Likewise, MCF10A cells show an uptake efficiency about 10-times less compared to their tumorigenic counterpart. These findings confirm that on average the capability of internalization and accumulation of GSH-NSs of the cancer cells is greater than the one of normal cells. Surprisingly, we observed that SKOV3 cells were not able to internalize the GSH-NSs, which accumulated outside the plasma membrane.

**Figure 3:**
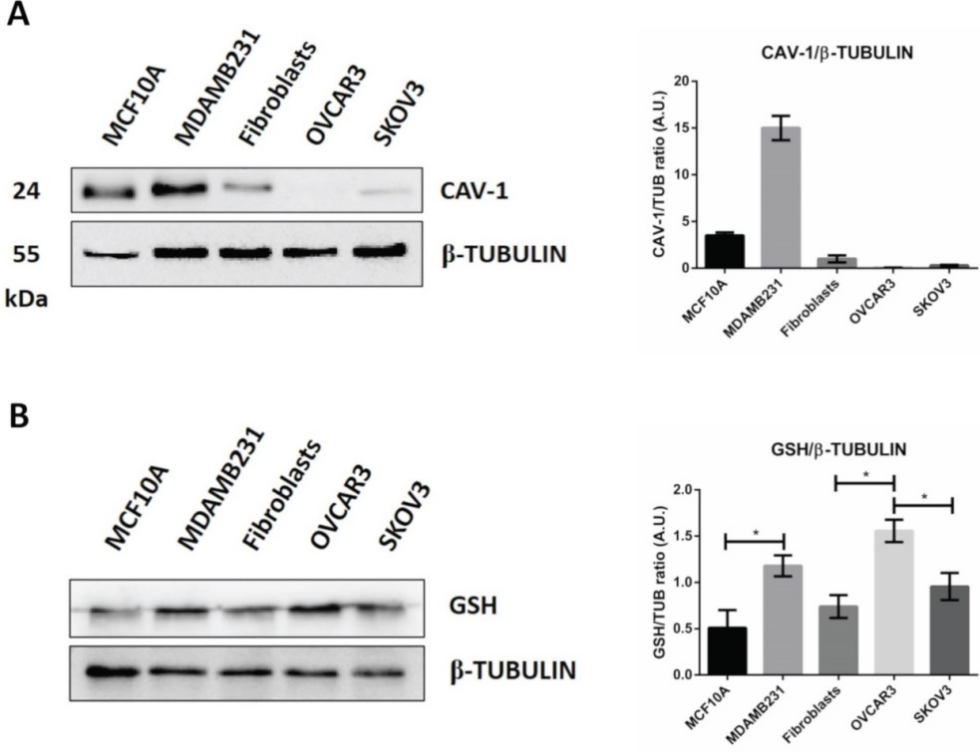
** GSH-NSs uptake is guided by intracellular GSH content.** (**A**) Western blotting showing the expression of caveolin-1. For loading control, the membranes were re-probed with β-Tubulin. Graph representing the densitometric analysis shown as average (A.U., arbitrary units) ±SD. (**B**) Western blotting to assess intracellular GSH content in cell models. For loading control, the membranes were re-probed with β-Tubulin. Graphs representing the densitometric analysis of three independent experiments are shown. Data are presented as average (A.U., arbitrary units) ±SD. Significance was considered as follows: *p < 0.05.

Caveolae-mediated endocytosis is one of the main routes for nanoparticle's cellular entry 42, 43, 44. We therefore asked whether caveolin-1 expression could explain the differential internalization process of GSH-NSs in the cell lines tested. The western blotting shown in Figure 3A suggests that GSH-NSs uptake is not dependent on caveolin-1-mediated endocytosis since OVCAR3 cells do not express this protein and yet can internalize the GSH-NSs, while SKOV3 cells that express caveolin-1 cannot.

GSH-NSs can be internalized and take advantage of disulfide bridges, which allow the release of the drug in the presence of high GSH concentration 36. Next, we validated that intracellular distribution and selective NSs delivery rely on the differences in the content of glutathione in the cell models used. As shown in Figure 3B, we found that OVCAR3 cells displayed the highest GSH content, followed by MDAMB231 and fibroblasts, and this was paralleled by the intracellular accumulation of NSs. SKOV3 cells failed to uptake the GSH-NSs despite exhibiting a GSH content similar to that in fibroblasts, suggesting that these cells may be deficient in some key factors involved in the mechanism of internalization of this particular material. On the other hand, MCF10A cells exhibited a modest GSH concentration, as expected for a non-tumorigenic cellular context. Thus, except for SKOV3 cells, the intracellular distribution profiles of β-CD-NSs observed above reflect their dependence on intracellular GSH content, which parallels the malignant phenotype.

### Resveratrol-mediated toxicity reflects the differential internalization of GSH-NSs in tumor cells

**Figure 4:**
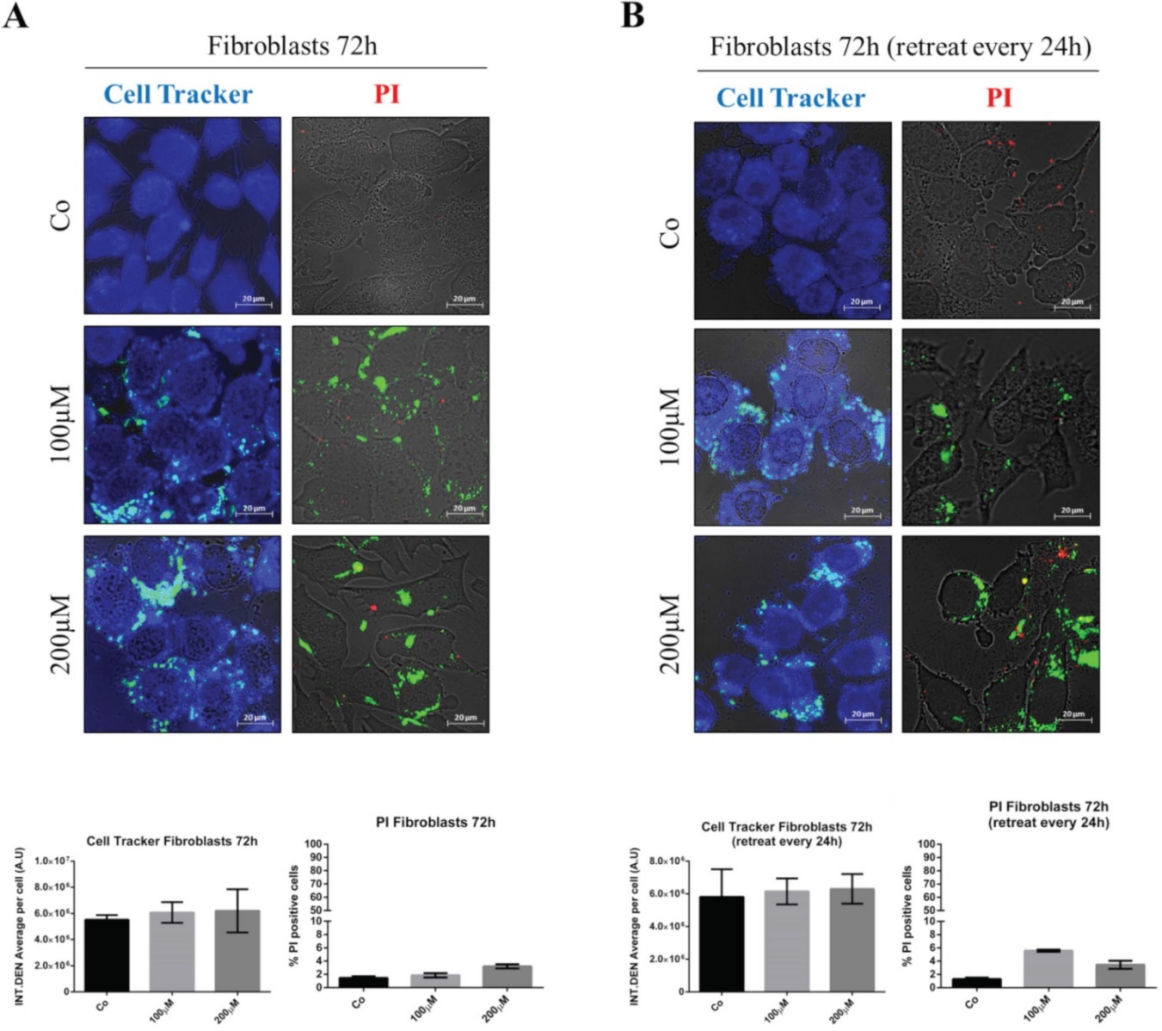
** Nude β-CD-GSH-NSs are not toxic for normal fibroblasts.** Fibroblasts were plated on sterile coverslips and treated for 72 hours with C6-GSH-NSs administered in single dose with no change of the medium (**A**) or in three doses (every 24 h) by renovating the treatment (**B**). Cells were labeled with Cell Tracker Blue fluorescent dye (left panel) or with Propidium iodide (PI) (right panel). Coverslips were washed and mounted on glasses and imaged immediately at the fluorescent microscope. Graphs represent the quantification of fluorescence intensity (INT.DEN. average per cell ± SD) for Cell Tracker staining, while cell death was assessed by counting the percentage of PI positive cells and represented in the graphs (% ± SD). Representative images of three independent experiments are shown.

First, we tested the potential toxicity of nude GSH-NSs toward normal human fibroblasts. The cells were exposed to the highest concentration and for up to 72 hours. Toxicity was imaged using Cell Tracker blue staining, which monitors mitochondrial functionality, and Propidium iodide, which labels the necrotic cells. As shown in Figure 4, no toxicity was observed when the fibroblasts were incubated with one single dose for 72 hours (panel A) nor when they were administered with three doses every 24 hours (panel B).

**Figure 5:**
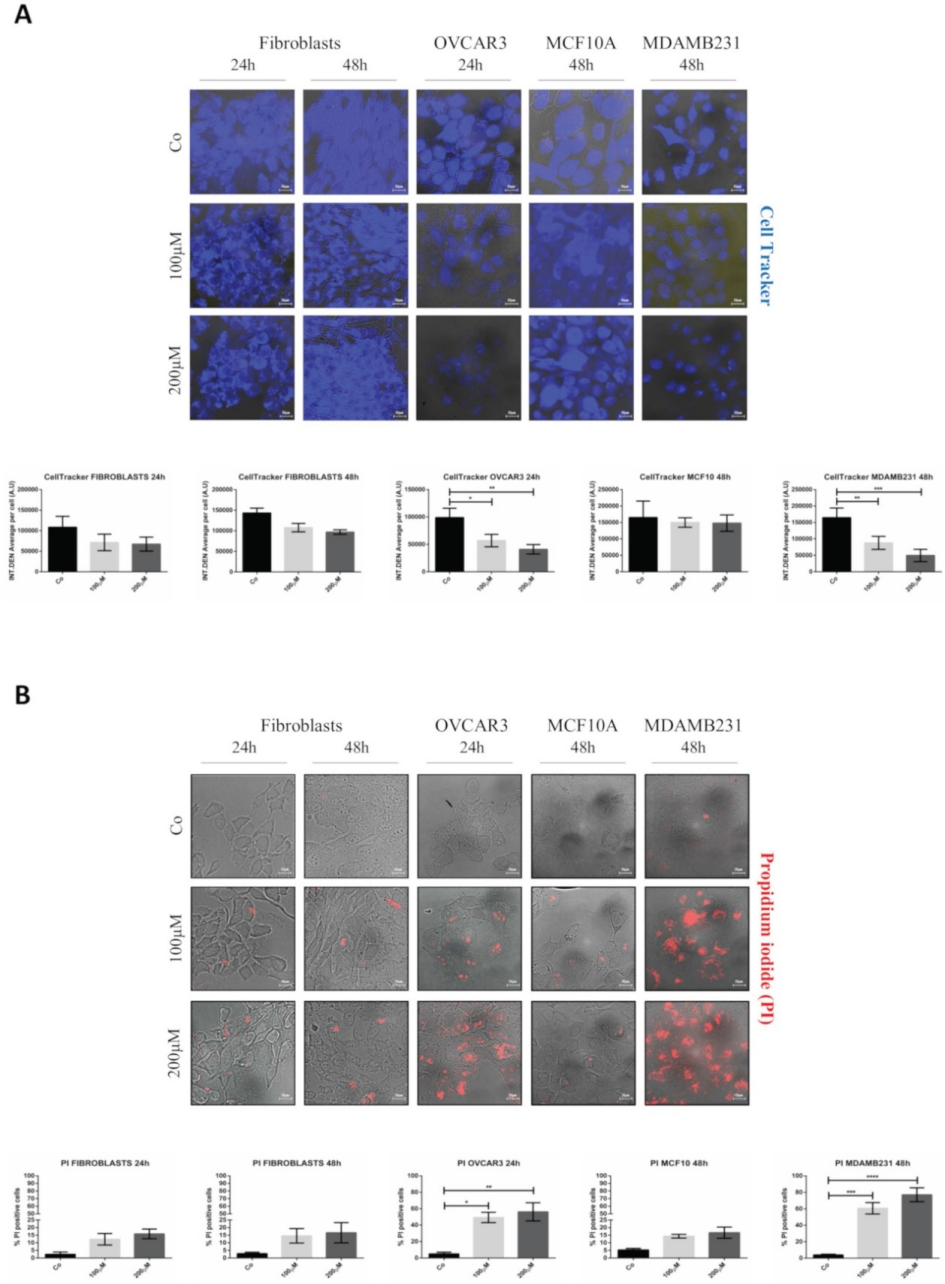
** RV-loaded-GSH-NSs selectively affect the cell viability of cancer cells compared to non-tumorigenic ones.** Cells were plated on sterile coverslips and treated with RV-GSH-NSs for the indicated time points. (**A**) Cells were labeled with Cell Tracker Blue fluorescent dye. Coverslips were washed and mounted on glasses and imaged immediately at the fluorescent microscope. Staining was performed in fibroblasts (24 and 48 hours), OVCAR3 (24 hours), MCF10A (48 hours) and MDAMB231 (48 hours). Graphs represent the quantification of fluorescence intensity (INT.DEN. average per cell) ± SD. Representative images of three independent experiments are shown. Significance was considered as follows: *p < 0.05; **p < 0.01; ***p < 0.001. (**B**) Cells were labelled with propidium iodide (PI). Coverslips were washed and mounted on glasses and imaged immediately at the fluorescent microscope. Staining was performed in fibroblasts (24 and 48 hours), OVCAR3 (24 hours), MCF10A (48 hours) and MDAMB231 (48 hours). Cell death was assessed by counting the percentage of PI positive cells and represented in the graphs (% ± SD). Representative images of three independent experiments are shown. Significance was considered as follows: *p < 0.05; **p < 0.01; ***p < 0.001; ****p<0.0001.

Next, we tested whether the delivery of RV by GSH-NSs effectively causes toxicity preferentially in tumor cells. The cells were exposed to two different concentrations of RV- GSH-NSs (100 and 200 µM) for 24 and 48 hours. Data show no significant difference in cell viability, as imaged by Cell Tracker, in fibroblasts and in benign epithelial MCF10A cells (Figure 5A), and in SKOV3 cells (data shown in Supplementary Figure S4), even when treated with the highest concentration for 48 hours.

In contrast, OVCAR3 and MDAMB231 cells show a dose-dependent reduction in cell viability, as indicated by the decreased Cell Tracker fluorescence intensity. This effect was more evident and anticipated in OVCAR3 compared to MDAMB231 cells. Taken together, these data show that RV-GSH-NSs preferentially affect cell viability in tumor cell lines compared to the non-tumoral ones, suggesting that the delivery and toxicity is (quite) selective for tumor cells expressing GSH at level much above the physiological level.

The selective toxicity of RV-GSH-NSs toward cancer cells was further confirmed by propidium iodide (PI) staining (Figure 5B). In this case, the increase of PI-positive cells (red fluorescent signal) indicates that cells undergo necrosis. In line with the previous results, cell death in fibroblasts and MCF10A cells was negligible, regardless of the time of incubation and concentrations of GSH-NSs. A similar pattern was observed in SKOV3 cells (data shown in Supplementary Figure S4). In contrary, OVCAR3 and MDAMB231 cells that express the highest level of GSH show a dose-dependent increase in cell death upon exposure to RV-GSH-NSs. Again, OVCAR3 cells show a higher sensitivity compared to MDAMB231 cells, consistent with the internalization rate.

Overall, these data confirm that the differential intracellular accumulation of the NSs between the cell lines that parallels the GSH content reflects in a selective toxic effect.

To determine whether toxicity resulted from apoptosis and/or necroptosis, we carried out a double staining with propidium iodide (PI) and annexin V-FITC (ANN-V) on living (not fixed) cells. We also employed the enzymatic inhibitors Necrostatin-1 (NEC-1) and ZVAD, that inhibit necroptosis and caspase-dependent apoptosis, respectively. As positive control to induce cell death in cancer cells, we employed the chemotherapeutic agent oxaliplatin (OxPt).

**Figure 6:**
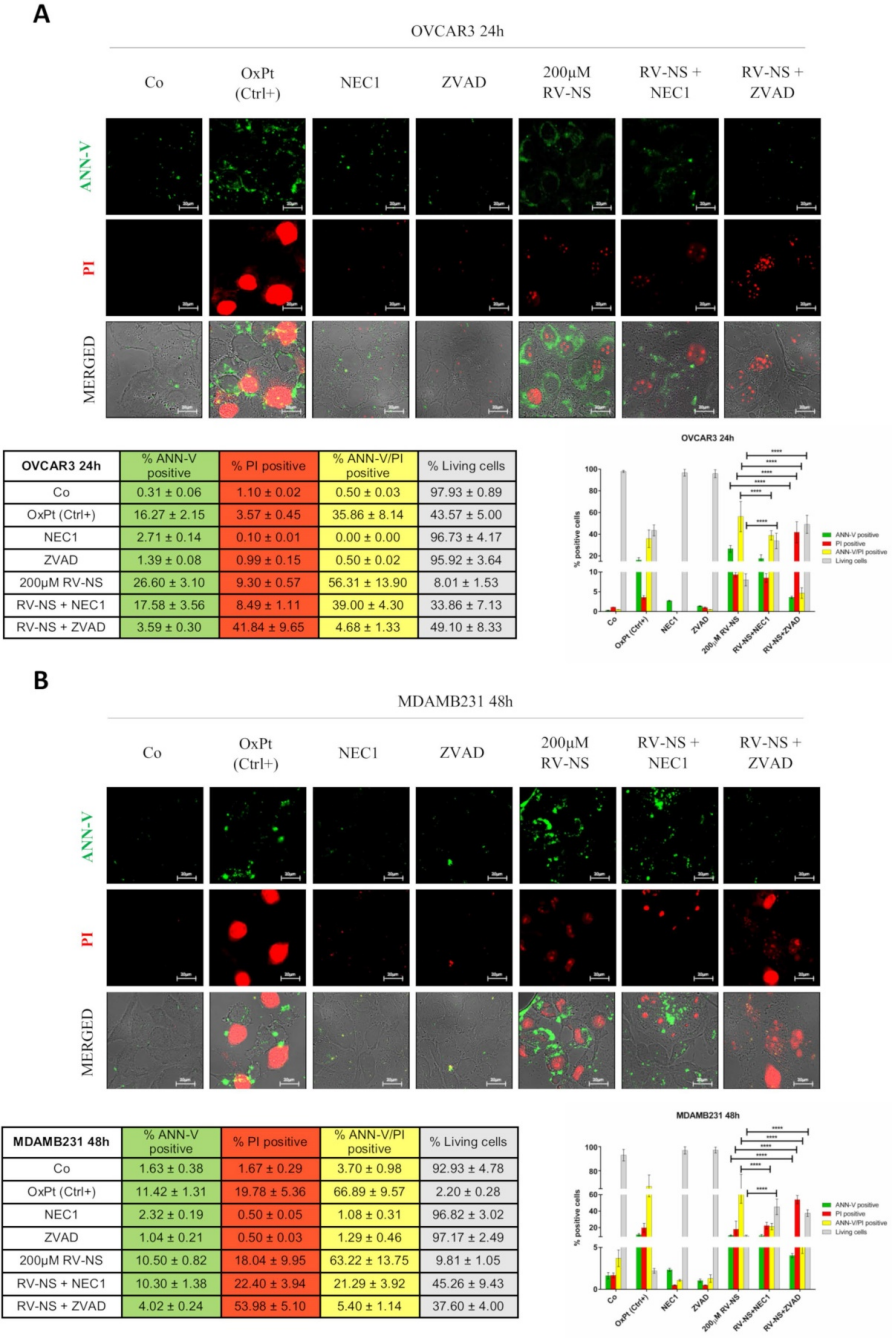
** RV-loaded-GSH-NSs promote an orchestrated mechanism of cell death that include caspase-dependent and caspase-independent pathways.** Cells were plated on sterile coverslips and treated with RV-GSH-NSs for the indicated time points: OVCAR3 cells for 24 hours (**A**) and MDAMB231 cells for 48 hours (**B**). Oxaliplatin was used as positive control, as it promotes cell death. To distinguish whether cell toxicity is associated to apoptosis or necroptosis, two inhibitors were employed: Necrostatin-1 (NEC1) and ZVAD, which inhibits primary necrosis and caspase-dependent apoptosis, respectively. Cells (not fixed) were double stained with Annexin V- FITC (ANN-V) and propidium iodide (PI) and coverslips were immediately imaged at the fluorescence microscope. Cell death was assessed by counting the percentage of ANN-V positive cells, PI positive cells, double positive and negative (living) cells. Data are presented as % ±SD and are reported in tables as well in bar graphs. Significance was considered as follows: ****p < 0.0001. All experiments have been reproduced at least three times in independent replicates.

We incubated OVCAR3 and MDAMB231 cells with the highest concentration of RV-GSH-NSs (200 µM) alone or in the presence of the two inhibitors. The representative images shown in Figure 6A indicate the presence of secondary necrosis (ANN-V/PI double positive) in OVCAR3 cells exposed to RV-GSH-NSs for 24 hours. Both the inhibitors could protect the cells from RV-GSH-NSs, yet to a different extent: NEC-1 increased by 3-folds and ZVAD by 5-folds the number of living (double-negative) cells, indicating that both apoptosis and necroptosis pathways are involved in the mechanism of toxicity.

In MDAMB231 cells incubated with RV-GSH-NSs for 48 hours about 70% display ANN-V/PI double positivity. Again, NEC-1 and ZVAD increased the proportion of living cells by 4.5-folds and 4.0-folds, respectively (Figure 6B).

To get a further insight on the mechanism of cell death, we assessed caspase 8 expression and cleavage by western blotting. In line with the previous findings, we observed only a slight activation of the caspase 8 cascade (Supplementary Figure S5). This suggests that RV-GSH-NSs trigger a caspase-independent necrotic cell death in parallel with the canonical apoptotic mechanism.

### GSH depletion strongly impairs RV-GSH-NSs intracellular accumulation in tumor cells

To finally prove that the targeting promoted by GSH-NSs was effectively guided by the intracellular concentration of glutathione, we depleted the cells of GSH by using buthionine sulfoximine (BSO), an inhibitor of gamma-glutamylcysteine synthetase (gamma-GCS).

**Figure 7:**
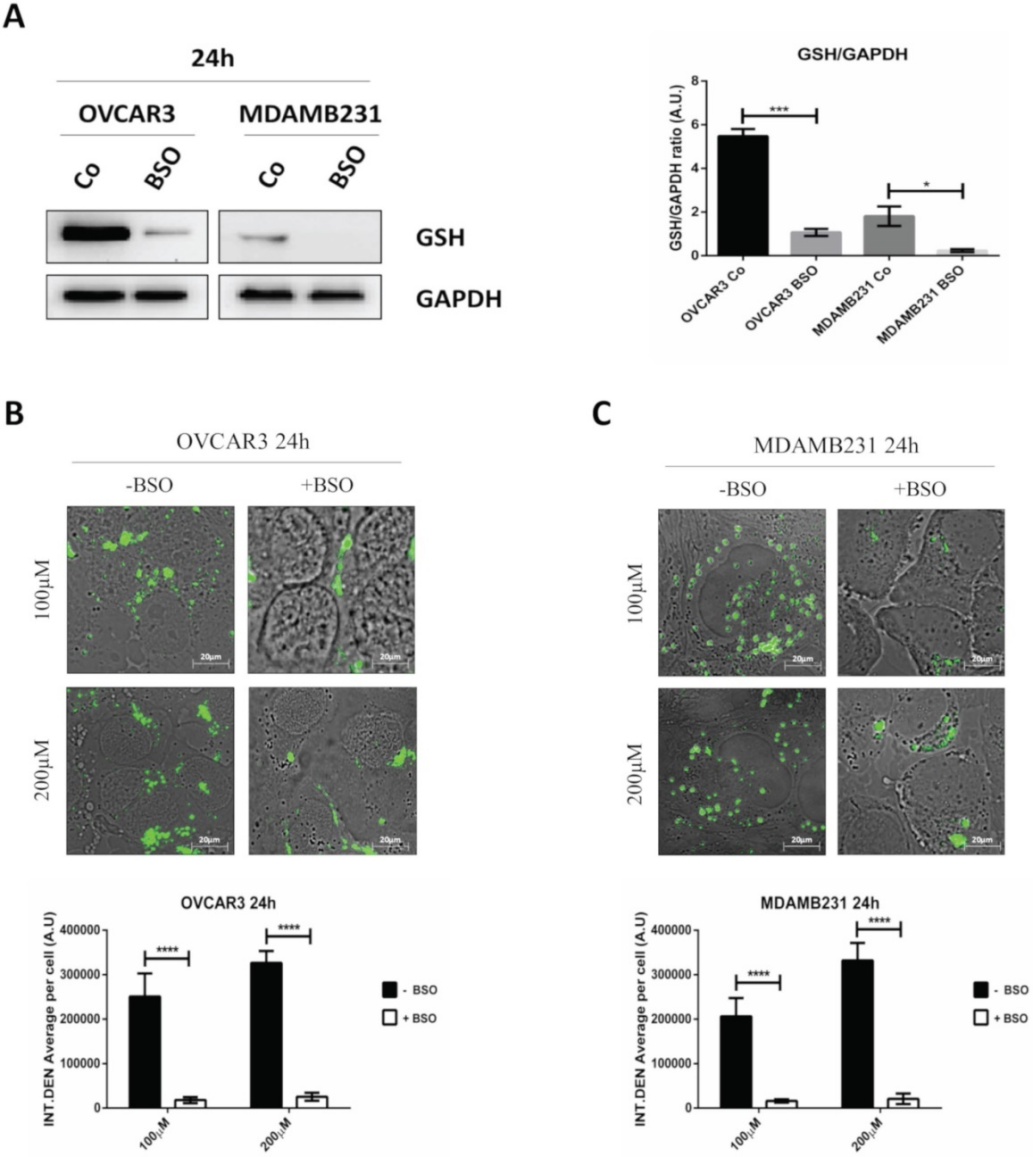
** GSH depletion by BSO strongly hampers the intracellular accumulation of GSH-NSs.** (**A**) Western blotting assessing the intracellular GSH content in OVCAR3 and MDAMB231 cells in the absence/presence of buthionine sulfoximine (BSO), an inhibitor of gamma-glutamylcysteine synthetase. For loading control, the membranes were re-probed with GAPDH. Graphs represent the densitometric analysis of three independent experiments. Data are presented as average (A.U., arbitrary units) ±SD. Significance was considered as follows: *p < 0.05; ***p < 0.001. (**B**-**C**) Cell were plated on sterile coverslips. Cell internalization studies were performed on OVCAR3 (**B**) and MDAMB231 cells (**C**) pre-treated or not with BSO and then incubated with C-6-GSH-NSs for 24 hours. Graphs representing the quantification of fluorescence intensity (INT.DEN. average per cell) ±SD are shown. Significance was considered as ****p < 0.0001.

To assay the efficiency of GSH depletion, we assessed GSH levels in OVCAR3 and MDAMB231 cells after the exposure to BSO for 24 hours. As shown in Figure 7A, BSO greatly reduced GSH expression in OVCAR3 cells and it completely suppressed GSH content in MDAMB231 cells.

The internalization rates of C-6-GSH-NSs in OVCAR3 and MDAMB 231 cells pre-treated or not with BSO is shown in Figure 7B-C. Both the cancer cells pre-treated with the inhibitor exhibited a significant decrease in the NSs intracellular accumulation at both concentrations considered (100 and 200 µM) after 24 hours of incubation. Moreover, upon treatment with BSO, the accumulation rate was similarly low for the two concentrations tested in both the cell models used, suggesting that GSH depletion strongly hampered the capability of cancer cells to redistribute GSH-NSs in the cytoplasm. The reduced intracellular accumulation of GSH-NSs does not result from NSs exocytosis and extrusion at late time, since the rate of accumulation of NSs in BSO-pre-treated cells is very low from the early beginning (30 minutes and 2 hours) (data in Supplementary Figure S6).

### GSH depletion prevents RV-GSH-NSs-induced toxicity in tumor cells

**Figure 8:**
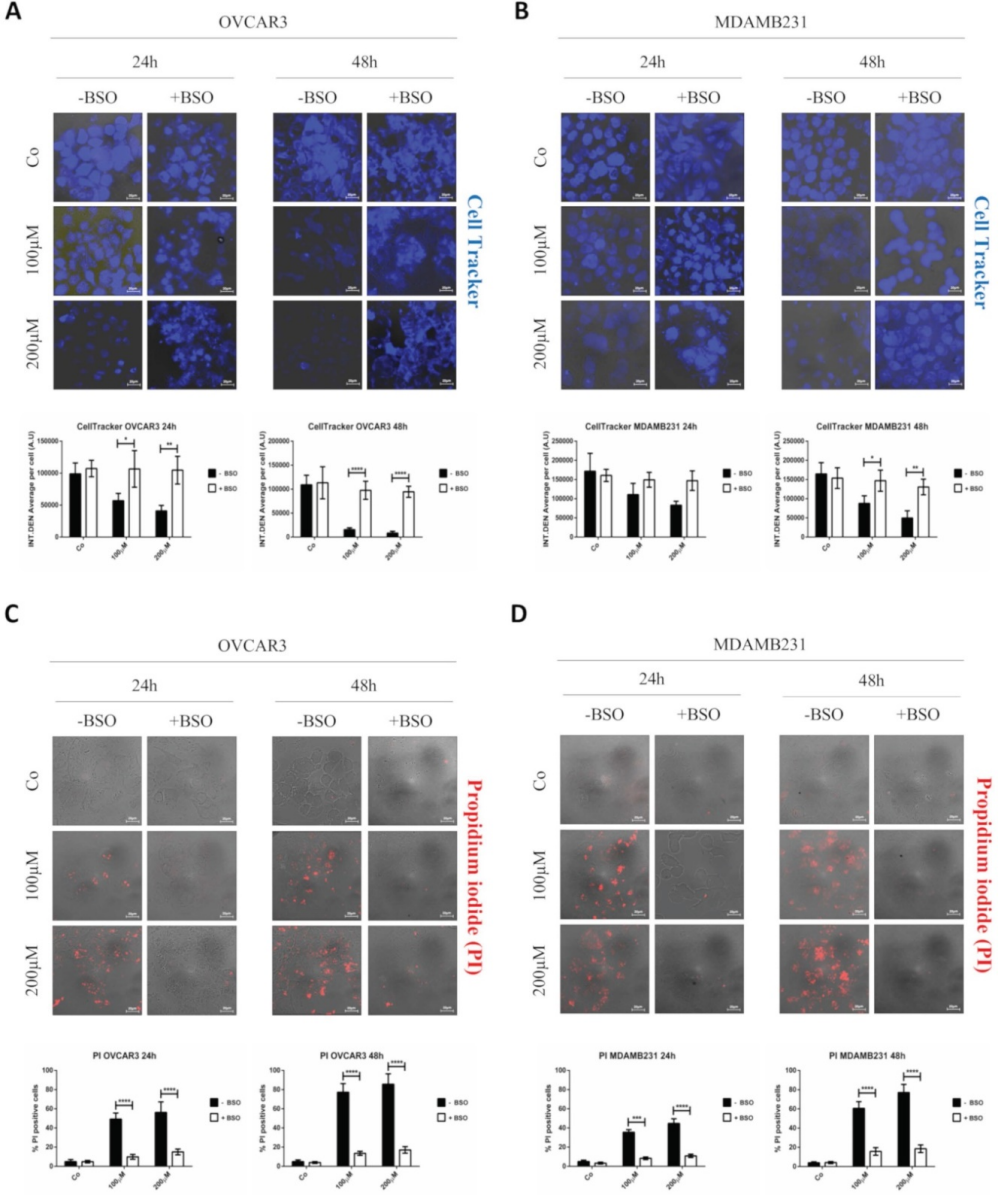
** GSH depletion by BSO prevents RV-mediated toxic effects in both cancer cell models.** Cells were plated on sterile coverslips, pre-treated or not with BSO and then incubated with RV-GSH-NSs for 24 and 48 hours. (**A**-**B**) Cells were labeled with Cell Tracker Blue fluorescent dye. Coverslips were washed and mounted on glasses and imaged immediately at the fluorescent microscope. Staining was performed in OVCAR3 (**A**) and MDAMB231 cells (**B**). Graphs represent the quantification of fluorescence intensity (INT.DEN. average per cell) ± SD. Representative images of three independent experiments are shown. Significance was considered as follows: *p < 0.05; **p < 0.01; ****p < 0.0001. (**C**-**D**) Cells were stained with propidium iodide (PI). Coverslips were washed and mounted on glasses and imaged immediately at the fluorescent microscope. Staining was performed in OVCAR3 (**C**) and MDAMB231 cells (**D**). Cell death was assessed by counting the percentage of PI positive cells and represented in the graphs (% ± SD). Representative images of three independent experiments are shown. Significance was considered as follows: ***p < 0.001; ****p<0.0001.

Finally, it was necessary to prove that cell toxicity induced by RV-GSH-NSs effectively relies on the presence of GSH for selective killing of cancer cells. Therefore, OVCAR3 and MDAMB231 cancer cells were pre-incubated with BSO for 16 hours and then exposed to RV- GSH-NSs. At the end, cell toxicity was assessed through Cell Tracker and propidium iodide staining. Compared to BSO-untreated cultures, the cell cultures depleted of GSH show a higher number of living cells (Cell Tracker positive) and a lower number of death cells (PI positive) upon exposure to RV-GSH-NSs (Figure 8). Taken together, these data demonstrate that GSH depletion by BSO results in a marked reduction of RV-mediated toxicity in both cancer cell models.

## Discussion

Nanotheranostics emerge for their potential application in biomedical field by combining multimodal imaging along with selective targeting therapy in the same nanoplatforms 45. Several nanocarriers have been proposed for the construction of novel theranostic agents including (i) liposomes 46, (ii) dendrimers 23, (iii) mesoporous silica nanoparticles 47, 48, (iv) gold nanoparticles 49, (v) super paramagnetic iron oxide nanoparticles 50, (vi) carbon nanotubes 51, and (vii) quantum dots 23.

Because of their safety and low toxicity, natural compounds have gained increasing attention in the prevention and treatment of cancer 52. Resveratrol, a naturally occurring polyphenol mainly found in many foods, such as mulberry, peanuts, grapes and red wine, exhibits chemopreventive and therapeutic effects on different cancers by targeting several molecules that play important roles in tumorigenesis 13. However, resveratrol exhibits a limited pharmacokinetic profile due to its poor bioavailability in the systemic circulation, since it is efficiently absorbed after oral administration but rapidly and extensively metabolized in both animal models and humans 53.

β-Cyclodextrin-based nanosponges (β-CD-NSs) may represent a promising option to enhance the therapeutic efficiency and bioavailability of poorly soluble molecules 37, 54, 55. CD-NSs have attracted increasing research attention thanks to the outstanding properties attributable to their peculiar structure. Notably, in addition to the biocompatibility typical of polysaccharides, this delivery system is characterized by the presence of tunable functional groups able to interact with biological tissues, making CD-NSs a good tool for targeted drug delivery 56.

Recently, our group developed glutathione-responsive β-CD-NSs loaded with drugs or imaging reporters (GSH-NSs). These innovative nanotools have been designed with the aim of selectively releasing anti-tumor drugs in cells with a high glutathione content, which represents the ideal internal stimulus for the rapid destabilization of nanocarriers and concomitant drug release within the target cells. When used in animals, pharmacokinetic and pharmacodynamic studies proved that these NS were safe and biodegradable with negligible toxicity, while they were efficiently targeted and highly toxic to the xenografted tumor 36.

Here we report that RV-loaded GSH-NSs selectively target and kill cancer cells by promoting cell toxicity that involved both apoptosis and necroptosis pathways. Additionally, RV can exert its anti-cancer activities by promoting autophagy-dependent cell death through the involvement of lysosomal cathepsin D 12, 14. Autophagy modulation could be exploited for therapeutic purposes, since autophagy has been reported to act both as an early pro-survival response and as a cell death mechanism after a chronic hyperstimulation. The differential response elicited depends on the genetic/epigenetic and metabolic status of the cancer cells targeted, thus underling the need of a critical assessment of these features when designing theranostics for personalized cancer therapy 40, 57. Further, the benefits promoted by RV include the interruption of pro-inflammatory cross-talk occurring within the tumor microenvironment 18, the inhibition of cancer cell migration 9 and the modulation of non-coding RNAs 10. In addition, RV can act as caloric restriction mimetic and could substitute for restriction diet by sensitizing cancer cells to chemotherapy along with the reduction of related side effects 11, 58.

Internalization of β-CD-NSs relies on different pathways of endocytosis which differs among the cell types 59. Here we found that RV-loaded GSH-NSs can target cancer cells regardless of their caveolin-1 expression. In fact, the NSs were internalized by OVCAR3 cells despite the absence of CAV-1 on their membrane. This aspect could represent an advantage for the application of CD-NSs, since CAV-1 is down-regulated in the vast majority of ovarian carcinomas 60, and its expression greatly change in different type of cancers, depending on tumor staging and the tumor microenvironment 61. On the other hand, these NSs could fail entering some tumors (as in SKOV3 cells in the present study) and this could represent a limitation for its therapeutic application. It seems that the redox status of the cell has an impact on the efficiency of the endocytosis of the β-CD-NSs, yet this process apparently is not dependent on caveolin-1. Further investigations are needed to address the reason of the differential entry of the GSH-responsive β-CD-NSs in the cancer cells. These data further outline the importance of assessing more in depth the molecular machinery involved in the uptake of β-CD-based NSs to support their employment in targetable cancers 62. In order to improve internalization, a possible breakthrough could be the functionalization of β-CD-NSs on their surface, thus allowing for exploitation of the specificity of receptor-mediated endocytosis 56.

Glutathione tends to be elevated in breast, ovarian, head and neck and lung cancers compared to the disease-free counterpart tissue, whereas its concentration is low in brain and liver tumors. Further, cervical, colorectal, gastric and esophageal cancers show heterogeneous levels of intracellular glutathione depending on the context 63. Thus, one possible weakness of our delivery system is related to the high heterogeneity of GSH content, that limits the application of these nanocarrier only to the tumors highly expressing GSH.

Interestingly, an increasing number of reports indicate that cancer-associated fibroblasts (CAFs) display high concentration of glutathione that can be released and transferred to cancer cells, pointing out that tumor GSH levels are finely-tuned by the dynamics occurring in the tumor microenvironment 64-67. Thus, the GSH-responsive delivery of RV could be exploited even in the modulation of the tumor stroma, allowing for targeting several culprits involved in cancer progression and chemoresistance. In this regard, next step will be to assess GSH in CAFs. Also, we will set a 3D co-culture of cancer cells and CAFs and check whether the RV -GSH-NSs can target both CAFs and cancer cells and whether it will diffuse through the layers and reach the most inner portion of the spheroids which mimics the microenvironment of the stem cell niche.

## Conclusions

In conclusion, GSH-NSs were developed to improve the solubilization of RV and selective targeting to the cancer cells without significant toxicity to normal cells. RV release was mediated by the intracellular GSH concentration which underlines the GSH-responsivity of the nanocarrier. These findings suggested that GSH-responsive nanocarrier can be used as an innovative tool for selective drug targeting in different types of cancers. Overall, our data demonstrate that RV-loaded GSH-NSs represent a valuable delivery system to target cancer cells by exploiting the differential tumor characteristics in terms of redox status in parallel with the limitation of side effects toward normal cells. In fact, physiological level of GSH in normal cells (here represented by skin fibroblasts and benign breast epithelial cells) seems not sufficient to trigger the release of the drug from the nanosponge.

## Highlights

- Stimuli-responsive nanosponges offer the advantage of site-specific drug delivery and release;
- Glutathione responsive nanosponges exploit the differential tumor characteristics in terms of redox status and limit the side effects toward normal cells;
- Resveratrol-loaded nanosponges are preferentially up-taken by cancer cells compared to non-tumorigenic cells;
- Resveratrol-loaded nanosponges selectively kill cancer cells.

## Supplementary Material

Supplementary figures.

## Abbreviations

RV: Resveratrol
β-CD: beta-cyclodextrin
PMDA: pyromellitic dianhydride
GSH: Glutathione
GSH-NSs: glutathione-responsive nanosponges
BSO: buthionine sulfoximine
