# Supplementary material for: Cyclodextrin nanosponge for the GSH-mediated delivery of Resveratrol in human cancer cells
Source: Nanotheranostics. 2021 Jan 21;5(2):197–212. doi: 10.7150/ntno.53888 (PMC7868003; doi:10.7150/ntno.53888)
Supplement: Supplementary file 1 — Supplementary figures. [file ntnov05p0197s1.pdf]

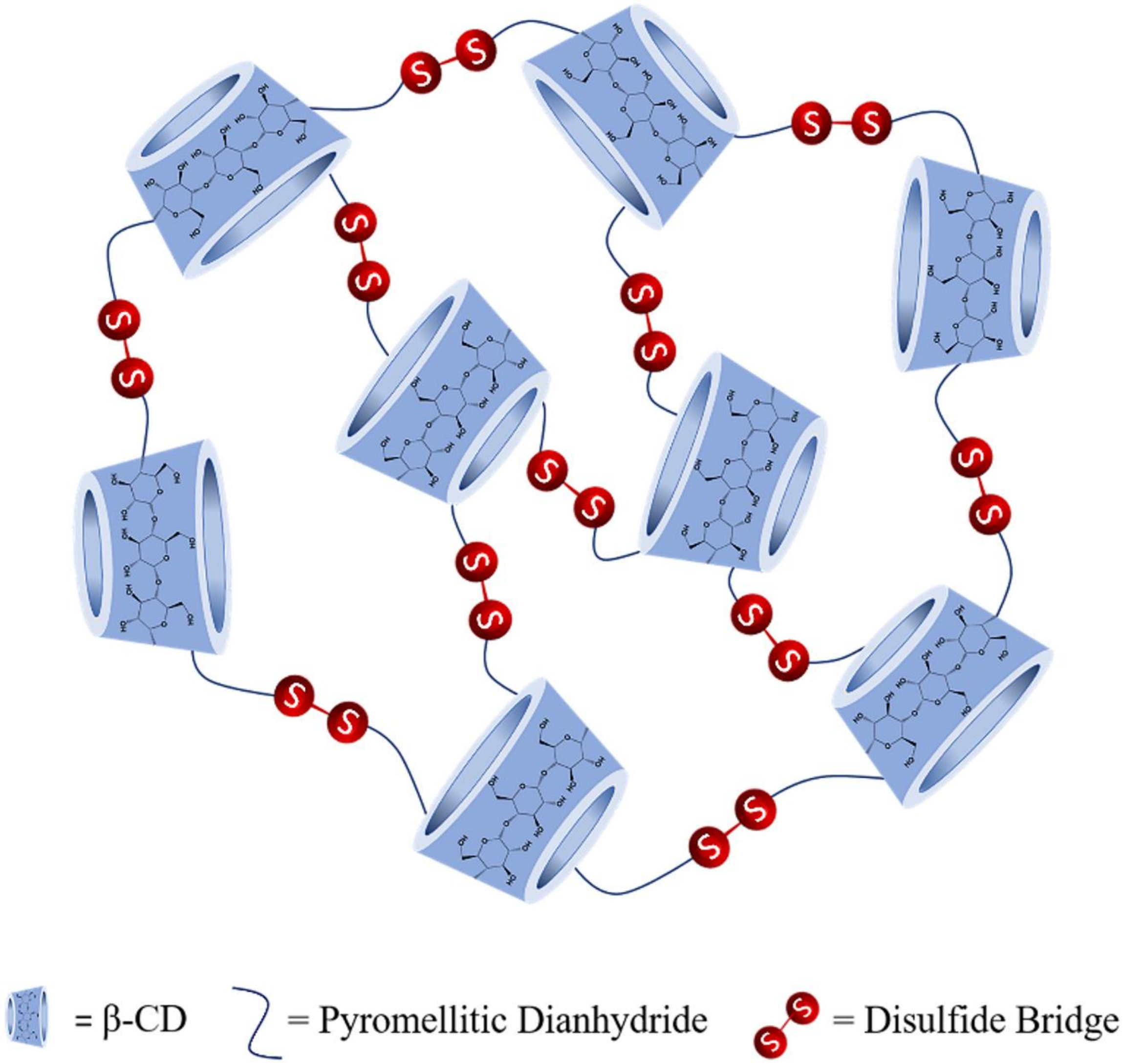

Supplementary Figure S1. Schematic representation of glutathione-responsive cyclodextrin nanosponges.

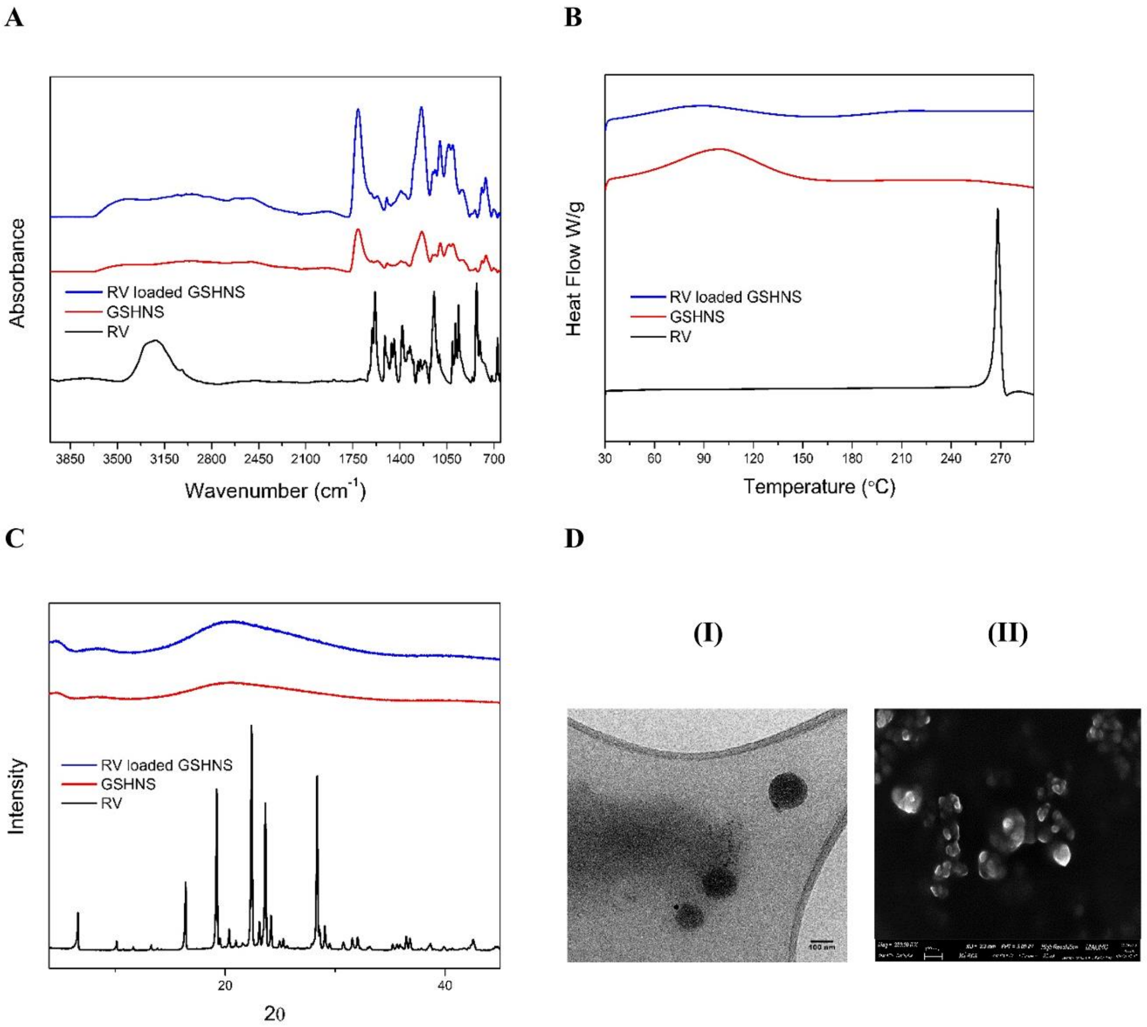

Supplementary Figure S2. Characterization of GSH-NS and RV-loaded GSH-NS.

(A) FTIR spectra of RV, RV-loaded GSH-NSs, and GSH-NSs. (B) DSC thermograms of RV, RV-loaded GSH-NSs, and GSH-NSs. (C) Diffraction pattern of RV, RV-loaded GSH-NSs, and GSH-NSs. (D) Morphology of the RV-loaded GSH-NSs (I) TEM and (II) FE-SEM.

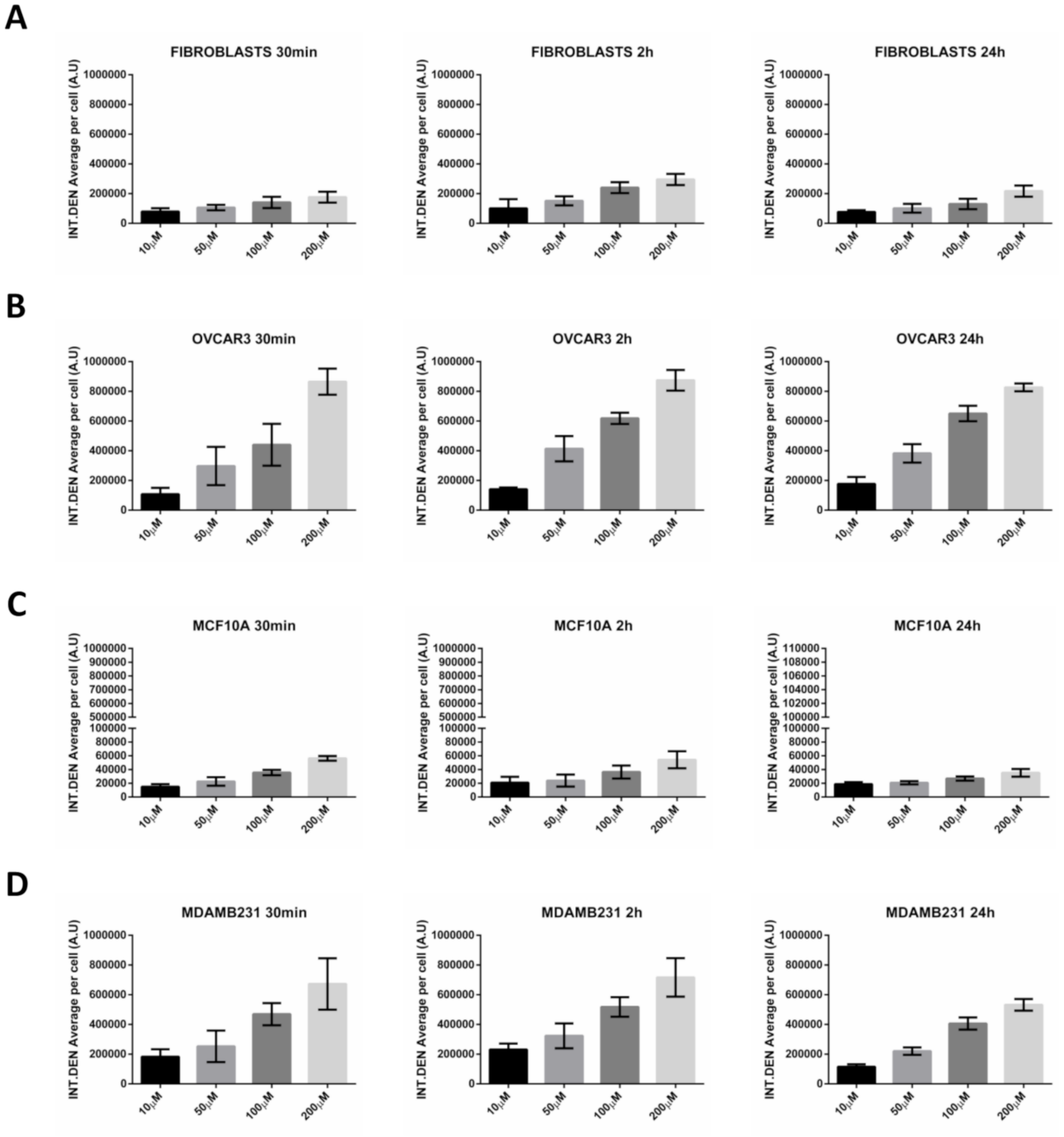

Supplementary Figure S3. Quantification of GSH-NSs entry.

Graphs representing the quantification of fluorescence intensity (INT.DEN. average per cell) ± SD referring to internalization studies reported in Figure 2. The experiments have been reproduced at least three times in independent replicates. Quantifications of GSH-NSs internalization were performed on normal fibroblasts (**A**), OVCAR3 cells (**B**), MCF10A (**C**) and MDAMB231 (**D**).

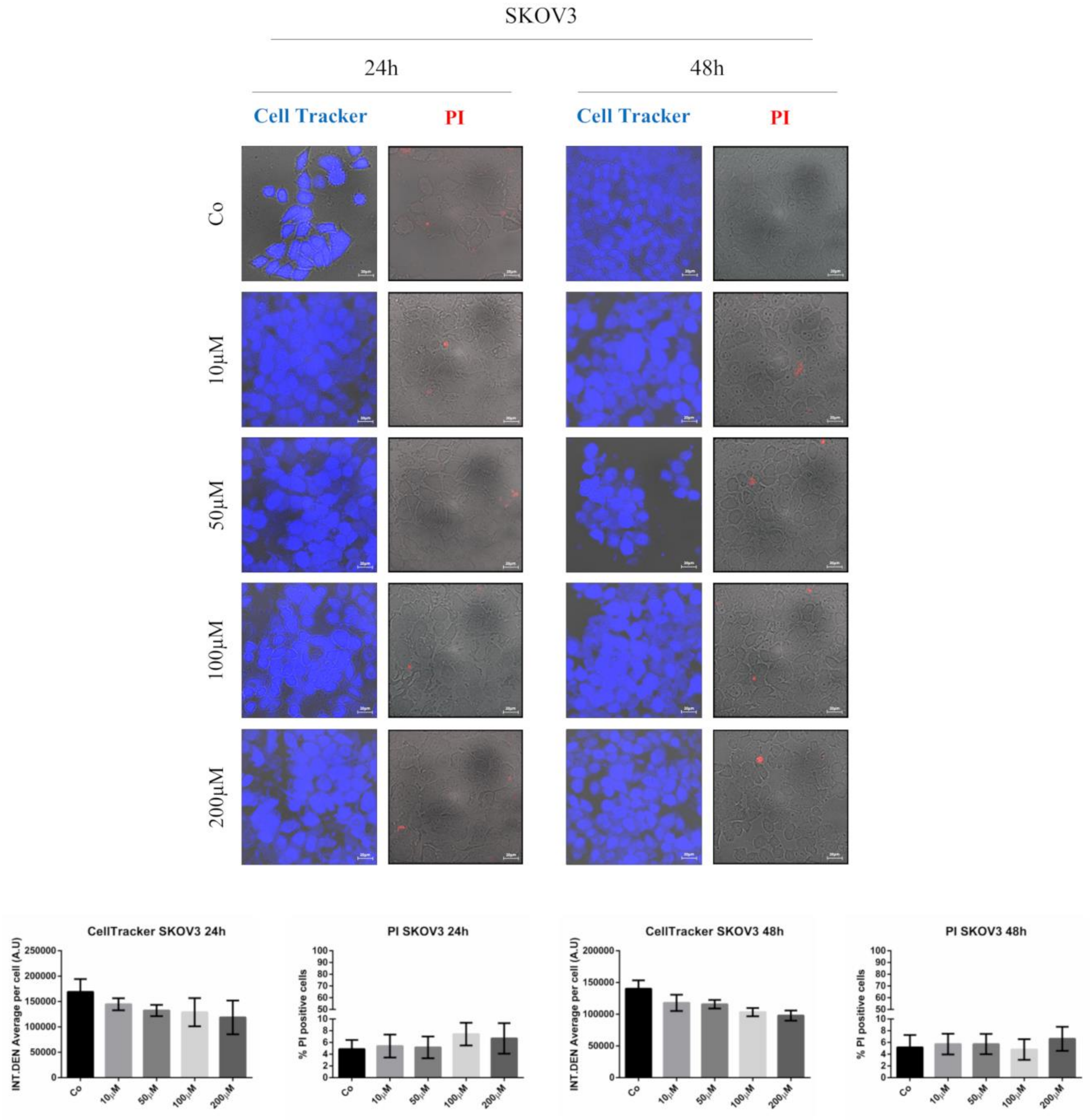

Supplementary Figure S4. RV-loaded-GSH-NSs do not alter SKOV3 cell viability.

Cells were plated on sterile coverslips and treated with increasing concentrations of RV-GSH-NSs for the indicated time points. Cells were labeled with Cell Tracker Blue dye and propidium iodide (PI). Coverslips were washed and mounted on glasses and imaged immediately at the fluorescent microscope. No significant effects on cell viability/toxicity were observed. This is in line with the limited internalization rates of GSH-NSs and lack of intracellular distribution observed in Figure 1C.

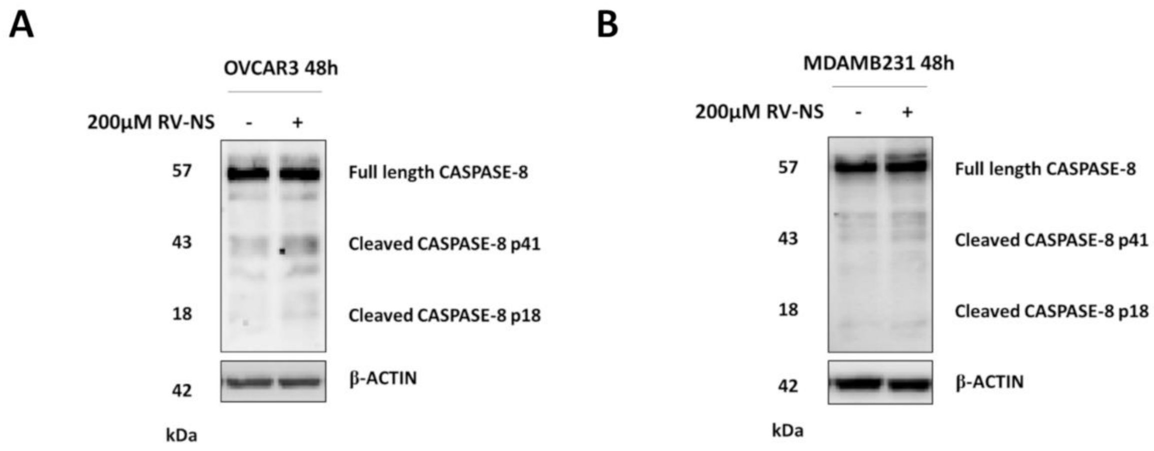

Supplementary Figure S5. RV-GSH-NS-mediated toxicity is mainly due to caspase-independent mechanisms.

Cells were plated on petri dishes and treated with the highest concentration of RV-GSH-NSs for 48 hours. Western blotting assessing the activation of caspase-8 cascade in OVCAR3 (**A**) and MDAMB231 cells (**B**). For loading control, the membranes were re-probed with β-Actin. We found only a slight activation of the caspase cascade, indicating that the toxicity elicited by RV-GSH-NSs involved mainly a caspase-independent cell death mechanism.

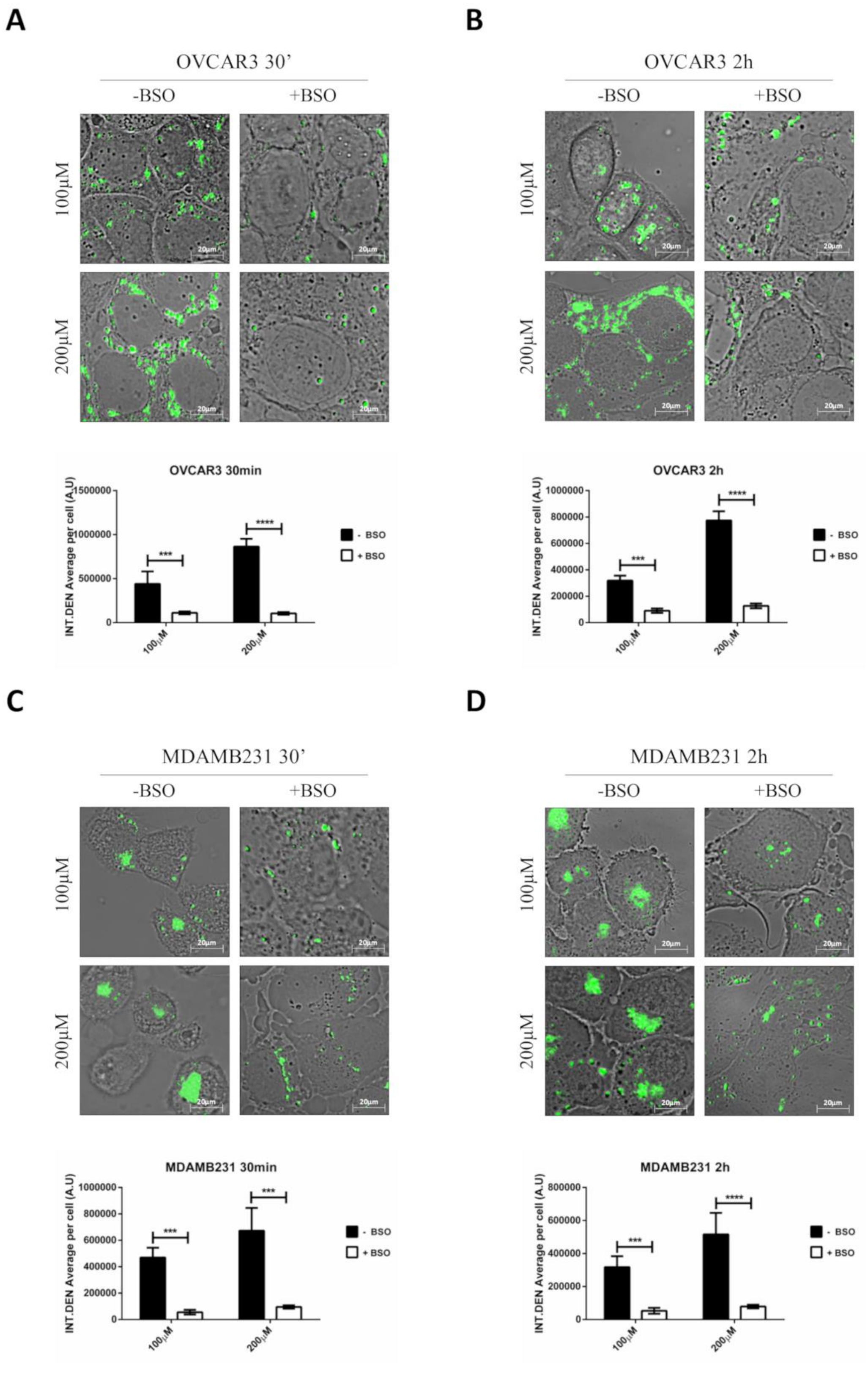

Supplementary Figure S6. BSO prevents the ability of GSH-NSs to target cancer cells.

Cells were plated on sterile coverslips. Cell internalization studies were performed in cells pre-treated or not with BSO. GSH-NSs uptake was monitored in OVCAR3 after 30 minutes (**A**) and 2 hours (**B**)

as well as in MDAMB231 cells after 30 minutes (**C**) and 2 hours (**D**). Data reflect the trend observed at 24 hours in Figure 5. Graphs representing the quantification of fluorescence intensity (INT.DEN. average per cell) ± SD. Significance was considered as follows: ***p < 0.001; ****p < 0.0001.
